# Informational needs and predictors of Jordanian breast and colorectal cancer survivors: a national cross-sectional study

**DOI:** 10.1007/s00520-022-07110-6

**Published:** 2022-05-10

**Authors:** Samar J. Melhem, Shereen Nabhani-Gebara, Reem Kayyali

**Affiliations:** Department of Pharmacy, Kingston University London, Penrhyn Road, Kingston, KT1 2EE England UK

**Keywords:** Jordan, Cancer survivor, Information, Middle East, Arab, Supportive care

## Abstract

**Purpose:**

To investigate the informational gap and predictors of information-seeking behaviour amongst survivors to inform survivorship planning and supportive cancer services for the population.

**Methods:**

A national cross-sectional survey of breast and colorectal cancer survivors was conducted in 2020 using a representative sample of those diagnosed in 2015/2016 as recruited from Jordan’s Cancer Registry (JCR). Participants responded to a 3-domain questionnaire: background information (*9 items*); information typologies (*13 items*) measured on a *5-*point Likert scale (*from very interested to extremely not interested*); timing of developing the information needs (*13 items*) (*ranging from immediately after diagnosis to after recurrence*). Logistic regression was used to determine the independent association between demographics and information-seeking behaviour amongst survivors. The chi-square test was employed to examine the association between categorical variables. ANOVA was used to compare the means of interest in cancer-related information between more than two groups.

**Results:**

Results show a relatively high overall information needs amongst survivors (3.68 ± 1.53). The most prevalent typologies were cancer staging (3.77 ± 1.593), treatment options (3.76 ± 1.55), and doctors’ communications (3.73 ± 1.62). ANOVA revealed no statistically significant differences between cancer types. 55.8% of patients desired information immediately after diagnosis and 23.3% developed their needs within 2 months. There was a statistically significant difference across all information typologies and educational attainment, age groups, monthly income, and employment (*P* < 0.05). Age was the only independent predictor of high information requirements amongst cancer survivors.

**Conclusion:**

Survivors reported high cancer information needs, suggesting that they may have been under-informed. Effective health communication programmes should be implemented to meet the informational needs.

**Supplementary Information:**

The online version contains supplementary material available at 10.1007/s00520-022-07110-6.

## Introduction

Breast cancer accounted for one in every eight malignancies diagnosed in 2020, with an estimated 2.3 million new cases. By 2020, further 684,996 breast cancer deaths are predicted, with a disproportionately high number occurring in low-resource settings. Meanwhile, colorectal cancer accounted for 1.15 million new cases and 576,858 deaths [1]. Breast cancer is the most common type of cancer in females in Jordan (38.5%), accounting for approximately 20.8% of all malignancies in both sexes; according to the National Registry (JCR), colorectal cancer ranked second in Jordan, affecting 10.9% [2]. Because of recent advancements in cancer treatment modalities and care quality, the life-altering burden of cancer survivorship has transitioned from the immediate consequences of anti-cancer treatment and survival rates towards a new spectrum of medical and non-medical challenges termed survivorship. Various definitions have been proposed to clarify ‘who is a cancer survivor’ [3, 4]. Both the National Coalition for Cancer Survivorship (NCCS) and the Institute of Medicine (IOM) agree that an individual is a ‘cancer survivor’ from the time of diagnosis until death. Therefore, cancer patients who are undergoing treatment are also referred to as ‘survivors of cancer’ [3, 4]. According to the EORTC’s Survivorship Task Force, a ‘cancer survivor’ is someone who has completed curative therapy and is presently disease-free or in remission [4]. Cancer survivorship encompasses the short- and long-term consequences of therapy, as well as adverse effects, secondary neoplasms, and psychosocial challenges [5, 6]. Many cancer survivors report that the adaptations required during survivorship are more challenging than those required during treatment. As a result, the healthcare sector is confronted with a significant challenge that necessitates the adoption of a patient-centred participatory approach that incorporates shared decision-making, self-management, patient empowerment, and the fulfilment of patients’ unmet information needs [7]. This approach can promote patient satisfaction and care quality, reduce anxiety about cancer and treatment, correct misconceptions and misinformation about cancer, improve adherence to treatment plans and patient-provider communications, and safeguard patients’ mental health and psychological well-being [7–10]. Patients typically seek information regarding their diagnosis, coping strategies, prognosis, and treatment choices, as well as cancer supportive care information [12]. Patient empowerment and realistic expectations regarding their conditions will assist them to cope well during the survivorship [8, 12]. Despite the substantial studies into cancer information needs and sources that have been conducted [11–13, 15–17], the studies have methodological limitations such as a homogeneous and unrepresentative population, small sample size, being exploratory in nature, or have low response rates [8–13, 16]. Additionally, cancer patients’ information-seeking patterns are influenced by clinical and socio-demographic factors (e.g. age, education, time since diagnosis) [14]. This limits generalizability and makes comparing patterns across cancer types difficult. Furthermore, the bulk of studies were conducted in western countries [13, 24], and other cultural contexts [15, 17, 21]. Yet, there is a paucity of research into the information needs of Arabs or Middle Eastern cancer survivors [18–20]. Consequently, it is crucial to assess non-western survivors’ preferences based on their socio-demographics and cultural identity to address their information requirements. This study sought to identify typologies and timings of information needs and their predictors amongst breast and colorectal cancer survivors to support effective survivorship planning and informational and supportive care services.

## Methods

### Study design and setting

A population-based cross-sectional survey was conducted between 1 March and July 17, 2020. The sample population was derived from all alive Jordanian breast and colorectal cancer survivors diagnosed in 2015–2016 who reside in Jordan and meet all the predefined eligibility criteria.

### Participants and recruitment

The study population was derived from the Jordan Cancer Registry (JCR) database in 2015–2016. The study population comprised 1567 adult survivors (≥ 18 years). Inclusion criteria include all the following: being a Jordanian citizen, participant was alive until 29/2/2020, and having correct contact details. Non-Jordanian citizens and those who were living abroad at the time of data collection or were unreachable because of missing contact information were excluded.

### Sampling procedure and randomisation

The Krejcie and Morgan equation determined a representative statistical sample size of 309 individuals [22]. The planned sample size was augmented by 30% (409) to account for anticipated non-response (due to death, rejection, error in phone number, etc.). Response proportions correspond to responded study sample/eligible sample size of population frame (*n*/*N*). To ensure adequate sample representation, the population was stratified into six layers according to cancer type and gender. Using SPSS (Statistical Package for Social Sciences) version 22, a systematic random sample was generated from the entire ranked population frame (1567) by age, gender, and type of cancer in ascending order. The first subject was chosen randomly from a table of random numbers, and the remaining subjects were chosen automatically using an explicit sampling frame according to a predetermined sampling interval (*k* = 4).

### Questionnaire

The study’s questionnaire was developed based on comprehensive review of the literature on cancer supportive care needs [11–13, 15, 17, 23–25]. Although there are various validated instruments related to the research topic, they are typically designed to measure unmet needs, contain few questions, or rate patient satisfaction [26]. The survey instrument was divided into three sections: background information, information needs typologies, and time of needs development since diagnosis. Section one consisted of 9 constructs that assessed the respondents’ socio-demographic factors, including age, gender, residence, marital status, employment, monthly income, education, comorbid diseases, and information sources. The second section, which included 13 constructs, examined the relationships between information typologies and patient interest since diagnosis. An ‘extremely uninterested’ in the typology of cancer information requirement since diagnosis was represented by a ‘1’ and ‘not interested’, ‘somewhat interested’, ‘interested’, and ‘very interested’ by a ‘2’, ‘3’, ‘4’, and ‘5’. The third section featured 13 constructs and compared the typology of cancer-related information needs to when patients developed those needs after diagnosis: ‘I developed that need immediately after diagnosis’, ‘1–2 months post diagnosis’, ‘upon completion of treatment’, ‘after recurrence’, or ‘I did not develop that need’. An additional blank space for Sects. 2 and 3 was provided to capture additional information. Overall ratings are aggregate scales and treated as continuous data [27]. Therefore, to determine the minimum and the maximum length of a 5-point Likert type scale, the range is calculated by 5 − 1 = 4 and then divided by five as it is the greatest value of the scale (4 ÷ 5 = 0.80). Afterwards, number one which is the least value in the scale was added in order to identify the maximum of this cell. Thus, very high interest in various information requirements was classified as 4.2–5; high 3.4–4.19; moderate 2.6–3.39; low 1.8–2.59; and low and very low 1.8–2.59 and 1–1.79, respectively.

### Pilot study and data quality assurance

#### Pilot study

In January 2020, the survey questionnaire was piloted. From January 7 to March 4, 2020, twenty-six (22 females and 4 males) ambulatory breast and colorectal cancer patients were recruited at the Jordan University Hospital (JUH), a semi-government tertiary hospital in the capital Amman. All Jordanian regions were represented with participants aged 28 to 79. Their educational and socioeconomic backgrounds were diverse; 61.5% of the 26 piloted had comorbid conditions. The pilot study assessed the questionnaire’s face validity, comprehensibility, and clarity of phrasing, as well as its length and format [28, 29]. The questionnaire was also reviewed by a senior oncology pharmacist and an oncologist. Because the survey instrument was originally developed in English, linguistic validation was required for the target Arabic-speaking audience. Forward/backward translation was used for linguistic and cultural validation [30]. Cronbach’s alpha was used to assess the internal consistency of the 26 responses. Statistical significance was set at 0.05. Cronbach alpha for Sects. 2 and 3 (13 subscales) was 0.995 and 0.999, respectively.

#### Data entry and analysis

Data were coded and entered on Excel sheets. The collected data was checked for accuracy and completeness. IBM SPSS 22 package was used for analysis. Descriptive statistics presented categorical data (percentages, frequency, mean, and standard deviation). The chi-square test was used to examine the association between categorical variables. The overall mean of Likert scale data was presented using mean and standard deviation. ANOVA was used to compare the means of interest in cancer-related information of more than two groups. Binary logistic regression model was used to determine independent predictors of information needs levels.

### Ethical considerations

Ethical approvals were obtained from Jordan’s Ministry of Health (MOH) approval number: MBA/ethics committee / 21,115 and Jordan University Hospital (JUH) approval number: 10/2019/8990 and the study was approved by Kingston University in accordance with the ethical requirements for scientific research (Approval Number: 2885). Prior to the interview, the researchers used a predesigned participant information sheet (PIS) to explain the study’s aims and objectives to each participant.

Completing the phone interview was considered implied consent to take part in the study. Participants’ confidentiality was guaranteed because they could not be identified as subjects and their data was only used to achieve the study’s objectives as per MOH and JCR permission.

## Results

### Response rate and socio-demographic characteristics of the respondents

The study included 335/409 with a response rate of 81.9% (Supplementary Table 1). Breast cancer response rate was 84.16%, colon cancer 73.8%, and rectal cancer 80.8% (Supplementary Table 2). Five patients (0.01%) died during data collection, 11 patients (1.45%) were unreachable owing to incorrect or disconnected phone numbers, and 2 patients (0.48%) were out of country during data collection (Supplementary Table 2).

Table 1 shows sample demographics: 83.9% (*n* = 281) of respondents were female, whilst 16.1% (*n* = 54) were male. The median age was 55 years (62.5 for males and 55 for females). 76.1% had breast cancer, 17.6% colon cancer, and 6.3% rectal cancer.

**Table 1:** Demographic characteristics of the sample (*N* = 335)

Socio-demographic characteristics	*n* (%)
Sex	
Male	54 (16.1))
Female	281 (83.9)
Age (in years)	
Less than 40	22 (6.6)
40–49	72 (21.5)
50–59	116 (24.6)
60–69	68 (20.3)
70 +	57 (17.0)
Cancer type	
Breast	255 (76.1)
Colon	59 (17.6)
Rectum	21 (6.3)
Regions	
North region	59 (17.6)
Middle region	264 (78.8)
South region	12 (3.6)
Marital status	
Single	19 (5.7)
Married	275 (82.1)
Divorced	3 (0.9)
Widowed	9 (2.7)
Refuse to answer	29 (8.7)
Employment status	
Employed (paid or unpaid)	57 (17.1)
Unemployed (capable or incapable)	6 (1.8)
Housewife	204 (60.9)
Student	0 (0.0)
Retired	68 (20.3)
Refuse to answer	0 (0.0)
Monthly income	
Less than 100 JD	16 (4.8)
100–199 JD (140–279$)	24 (7.2)
200–299 JD (280–419$)	42 (12.5)
300–499 JD (420–699$)	63(18.8)
500 JD (700$) or more	62 (18.5)
Do not know	77 (23.0)
Refuse to answer	51 (15.2)
Education status	
Illiterate	26 (7.8)
Elementary school	78 (23.3)
High school (Tawjihi)	91 (27.2)
Diploma	67 (20.0)
University /bachelor’s degree	63 (18.8)
Masters/PhD	10 (3.0)
Comorbid conditions	
Diabetes	75 (22.7)
Hypertension	110 (33.2)
Cardiovascular disease	29 (8.8)
Other	25 (7.6)
No chronic diseases	175 (52.9)

**Fig. 1:**
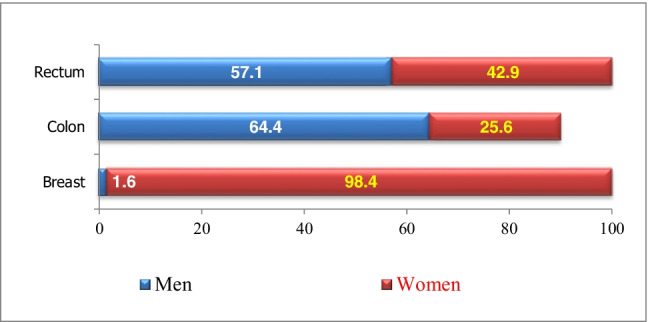
Distribution of the respondents by gender and type of cancer, Jordan 2020

Figure 1 depicts sample gender and cancer type distribution. 72.5% of female respondents were housewives. The second most common employment status was retired (20.3%), followed by self-employed (17.1%). The majority (61.2%) lived in the capital Amman and were married (82.1%). 38.2% of patients refused to answer or said ‘don’t know’ when asked about monthly household income. The monthly income for 43.3% was under $750 and 18.5% reported monthly income over $750. 33.2% of cancer survivors had hypertension, 22.7% diabetes, and 8.8% cardiovascular disease. Only 21.8% had a graduate degree, 27.2% had a high school diploma, 20.0% had an elementary education, and 7.8% were illiterate.

### Information sources and trends for health-related information

Patients were asked to identify their cancer information sources (Supplementary Table 3). Patients (85.1%) rely on their doctor for information, followed by the internet (46.6%). Third place went to family, friends, and peer survivors’ recommendations (11.9%). However, only 6.9% of patients sought professional counselling (e.g. nurses and pharmacists).

### Typology and timing of information needs acquisition for breast and colorectal cancer survivors

Since diagnosis, the aggregate level of interest (mean ± SD) in various cancer information needs is shown in Table 2. The overall interest in cancer needs was high (3.68 ± 1.53). Breast cancer (3.69 ± 1.51), colon cancer (3.64 ± 1.60), and rectal cancer (3.71 ± 1.53) had slightly higher than average level of interest. Patients’ most frequently expressed requirements were for information on disease stage, progression and recurrence (3.77 ± 1.59), treatment options (3.76 ± 1.62), and communication with clinicians (3.73 ± 1.62), whilst information on insurance and financial costs (3.37 ± 1.76) were least desired.

**Table 2:** Average level of interest in cancer-related information typologies since the time of diagnosis for breast and colorectal cancer patients (*N* = 335)

	Typology of cancer information need(s)	Mean	SD
1	Information about available and best treatment options	3.76	1.62
2	Information about the disease itself (breast/colon)	3.71	1.62
3	Treatment-related adverse effects, lab test, ultrasound, late effects of treatment	3.74	1.61
4	Disease staging, the likelihood of progression and recurrence	3.77	1.59
5	Home care activities post-surgery and between cycles of chemotherapy	3.72	1.61
6	Information about communicating with medical teams and contacting consultants	3.73	1.62
7	Effect of cancer on family, work, relationships	3.71	1.63
8	Physical image, sexuality/fertility	3.71	1.63
9	Psychological, emotional, and coping strategies	3.66	1.66
10	Nutritional plans, diet, and physical activity	3.66	1.66
11	Secondary prevention (mammography, colonoscopy, self-examination, lifestyle modification)	3.70	1.63
12	Information about insurance and financial costs	3.37	1.76
13	Information about medications for cancer and other comorbid conditions (e.g. heart disease, diabetes)	3.65	1.66

One-way ANOVA test with post hoc analysis was used to assess the difference or the variation of cancer information typologies and type of cancer and socio-demographic characteristics. There was no statistically significant difference between cancer groups as determined by one-way ANOVA (*F* = 0.032, *P* = 0.968) (Table 3). Patients who were employed expressed a higher degree of interest in the overall cancer-related information typologies (4.22 ± 1.94) than those who were unemployed (2.06 ± 0.99) or housewives (3.51 ± 1.61), respectively, and that was found to be statistically significant (*F* = 6.316, *P* = 0.000) (Table 3). Cancer-related information requirements were also substantially higher amongst patients with higher monthly income of > 500 JD (4.39 ± 1.09), compared to low-income individuals (3.18 ± 1.75). Furthermore, a significant statistical difference was found (*F* = 8.46, *P* = 0.00) for patients who hold Masters/PhD whereby they exhibited the highest desire for cancer information needs (4.46 ± 0.99) in comparison to illiterate patients (2.41 ± 1.71).

**Table 3:** Test of statistical significance (one-way ANOVA) of the variation in the overall mean level of interest on cancer-related information by socio-demographics variables

Variables	Overall interest in cancer-related information typologies (*n* = 13) since the time of diagnosis, mean (SD)	*F* value	Sig
Type of cancer		0.032	0.968
Breast	3.69 (1.51)		
Colon	3.64 (1.60)		
Rectum	3.71 (1.55)		
Total mean (SD)	3.69 (1.53)		
Age (in years)		10.327	0.000
< 40	4.33 (1.00)		
40–49	4.07 (1.32)		
50–59	3.822 (1.42)		
60–69	3.72 (1.57)		
≥ 70	2.62 (1.65)		
Total mean (SD)	3.69 (1.53)		
Marital status		4.203	0.002
Single	4.27 (1.55)		
Married	3.76 (1.48)		
Divorced	2.33 (2.31)		
Widowed	3.54 (1.39)		
Refused to answer	2.77 (1.79)		
Total mean (SD)	3.68 (1.53)		
Residence		2.074	0.127
North region	3.74 (1.50)		
Middle region	3.36 (1.68)		
South region	4.15 (1.22)		
Total mean (SD)	3.69 (1.53)		
Education		8.461	0.000
Illiterate	2.41 (1.71)		
Elementary school	3.25 (1.64)		
High school (Tawjihi)	3.90 (1.47)		
Diploma	3.74 (1.45)		
University degree	4.27 (1.04)		
Masters/PhD	4.46 (0.99)		
Total mean (SD)	3.69 (1.53)		
Employment		6.316	0.000
Employed (paid or unpaid)	4.22 (1.14)		
Unemployed (capable or incapable)	2.06 (0.99)		
Housewife	3.51 (1.61)		
Student	0.00 (0.0)		
Retired	3.91 (1.42)		
Refused to answer	0.00 (0.0)		
Total mean (SD)	3.68 (1.53)		
Monthly income (JD)		3.691	0.001
< 100 JD	3.18 (1.75)		
100–199 JD	3.20 (1.69)		
200–299 JD	3.46 (1.69)		
300–499 JD	3.80 (1.58)		
500 JD or more	4.39 (1.09)		
Don’t know	3.41 (1.55)		
Refused to answer	3.69 (1.36)		
Total mean (SD)	3.69 (1.53)		

The findings indicated a significant difference across all information typologies based on educational level, and age groups (*P* < 0.05). Also, there were significant variation based on monthly income and employment status, for cancer-related information domains, except for financial information and insurance domain for employment status (*P* = 0.442) and monthly income (*P* = 0.134). The result indicated no significant difference based on gender, cancer type, and residential area for cancer information typologies (Table 4).

**Table 4:** Mean score of level of interest in cancer-related information typologies by socio-demographic characteristics, Jordan 2020, *p* value * <0.05 indicates statistical significance

Demographic variables	Treatment options	Disease-specific information	Treatment-related adverse effects, lab test, ultrasound, late effects of treatment	Disease staging, progression, and recurrence	Post-operative homecare activities and between chemotherapy	Communication with medical teams	Cancer impact on relationships	Physical image, sexuality, fertility	Nutritional plans	Psychological, coping, and emotional strategies	Secondary prevention	Insurance and financial costs	Medication plans and comorbidities
Gender													
Female	3.70 (1.63)	3.69 (1.64)	3.73 (1.62)	3.75 (1.61)	3.89 (1.51)	3.69 (1.64)	3.69 (1.64)	3.68 (1.64)	3.64 (1.66)	3.64 (1.67)	3.70 (1.64)	3.33 (1.77)	3.63 (1.67)
Male	3.81 (1.56)	3.81 (1.56)	3.81 (1.56)	3.87 (1.53)	3.69 (1.63)	3.93 (1.52)	3.85 (1.56)	3.85 (1.56)	3.72 (1.64)	3.80 (1.61)	3.72 (1.64)	3.57 (1.70)	3.76 (1.65)
*P* value	.550	.420	.583	.532	.247	.212	.412	.430	.609	.932	.765	.365	.877
Age (in years)													
Less than 40	4.18 (1.37)	4.14 (1.46)	4.09 (1.51)	4.55 (1.01)	4.50 (1.10)	4.14 (1.26)	4.41 (1.26)	4.64 (0.95)	4.32 (1.29)	4.38 (1.33)	4.59 (1.01)	3.68 (1.76)	4.45 (1.22)
40–49	4.19 (1.46)	4.11 (1.47)	4.18 (1.42)	4.18 (1.40)	4.06 (1.49)	4.07 (1.48)	4.15 (1.47)	4.06 (1.51)	4.07 (1.51)	4.11 (1.49)	4.10 (1.48)	3.67 (1.68)	4.00 (1.54)
50–59	3.87 (1.54)	3.87 (1.51)	3.92 (1.49)	3.92 (1.50)	3.88 (1.51)	3.88 (1.51)	3.88 (1.51)	3.83 (1.55)	3.81 (1.57)	3.78 (1.60)	3.87 (1.52)	3.45 (1.75)	3.78 (1.58)
60–69	3.81 (1.61)	3.76 (1.62)	3.78 (1.58)	3.76 (1.60)	3.75 (1.62)	3.75 (1.62)	3.75 (1.62)	3.75 (1.62)	3.69 (1.64)	3.69 (1.64)	3.69 (1.64)	3.50 (1.72)	3.74 (1.62)
70 +	2.77 (1.73)	2.65 (1.70)	2.65 (1.70)	2.63 (1.70)	2.65 (1.70)	2.68 (1.72)	2.68 (1.72)	2.61 (1.68)	2.53 (1.68)	2.60 (1.70)	2.53 (1.68)	2.58 (1.73)	2.53 (1.72)
*P* value	.000*	.000*	.000*	.000*	.000*	.000*	.000*	.000*	.000*	.000*	.000*	.004*	.000*
Marital status													
Single	4.16 (1.50)	4.21 (1.51)	4.05 (1.51)	4.37 (1.30)	4.37 (1.30)	4.37 (1.30)	4.37 (1.30)	4.32 (1.29)	4.32 (1.29)	4.32 (1.29)	4.37 (1.07)	4.11 (1.33)	4.21 (1.32)
Married	3.84 (1.57)	3.80 (1.57)	3.84 (1.55)	3.84 (1.54)	3.79 (1.57)	3.81 (1.57)	3.78 (1.59)	3.78 (1.59)	3.72 (1.62)	3.73 (1.62)	3.78 (1.60)	3.44 (1.74)	3.72 (1.63)
Divorced	2.33 (2.31)	2.33 (2.31)	2.33 (2.31)	2.33 (2.31)	2.33 (2.31)	2.33 (2.31)	2.33 (2.31)	2.33 (2.31)	2.33 (2.31)	2.33 (2.31)	2.33 (2.31)	2.33 (2.31)	2.33 (2.31)
Widowed	2.93 (1.91)	2.69 (1.85)	2.83 (1.87)	2.83 (1.87)	2.83 (1.87)	2.83 (1.87)	2.83 (1.87)	2.83 (1.87)	2.83 (1.87)	2.69 (1.85)	2.69 (1.85)	2.45 (1.76)	2.83 (1.87)
Separated	3.67 (1.5)	3.67 (1.5)	3.67 (1.5)	3.67 (1.50)	3.67 (1.50)	2.22 (1.64)	3.67 (1.50)	3.67 (1.50)	3.33 (1.73)	3.67 (1.50)	3.67 (1.50)	3.11 (1.76)	3.33 (1.73)
*P* value	.018*	.002*	.009*	.003*	.005*	.003*	.006*	.008*	.004*	.011*	.002*	.011*	.017*
Residence													
North region	3.82 (1.59)	3.78 (1.59)	3.80 (1.58)	3.82 (1.56)	3.78 (1.58)	3.78 (1.59)	3.76 (1.60)	3.78 (1.59)	3.70 (1.63)	3.71 (1.63)	3.73 (1.62)	3.43 (1.74)	3.69 (1.65)
Middle region	3.36 (1.73)	3.29 (1.78)	3.39 (1.75)	3.42 (1.74)	3.42 (1.74)	3.41 (1.74)	3.42 (1.74)	3.42 (1.74)	3.31 (1.78)	3.36 (1.76)	3.44 (1.76)	3.10 (1.81)	3.31 (1.76)
South region	4.42 (1.24)	4.33 (1.30)	4.33 (1.30)	4.33 (1.30)	3.92 (1.68)	4.08 (1.44)	4.17 (1.59)	3.64 (1.80)	4.42 (1.24)	4.08 (1.56)	4.25 (1.29)	3.42 (1.88)	4.42 (1.24)
*P* value	0.05	0.04*	0.09	0.10	0.28	0.20	0.22	0.32	0.22	0.07	0.23	0.43	0.07

Approximately 55.8% of survivors desired information immediately after diagnosis, whilst 23.3% developed their requirements 2 months later. Compared to men, over two-thirds of women acquire information needs immediately after diagnosis. During recurrence, survivors’ information seeking almost diminished. Also, 16% of patients had not developed information needs at any particular time.

### Factors influencing information-seeking behaviour

Binary logistic regression analysis indicated age as an independent predictor of high information-seeking pattern; survivors under 40 years of age were 110.5% more likely than 70 + years counterparts to have a high degree of interest in obtaining cancer information (AOR 2.105, CI = (1.933–34.838)). Survivors aged 40–49 were 71.9% more likely than those aged 70 + to have high or very high interest in overall information typologies (AOR 1.719, CI = (2.302–13.516)). Survivors aged 50–59 are 31.4% more likely than those aged 70 + (AOR = 1.314, CI = (1.735–7.986)). Also, those aged 60–69 are 29.1% more likely to have higher information needs compared to 70 + (AOR = 1.291, CI = 1.604–8252)) (Table 5). None of the other variables was shown to be significant predictors of high information need of survivors.

**Table 5:** Predictors of the overall level of information needs amongst breast and colorectal cancer survivors, Jordan 2020, *p*-value * <0.05 indicates statistical significance

	Information-seeking behaviour as per degree of interest in cancer information needs				
Variables	Very low to moderate level of interest in cancer information level; 1 (*n* = 118)	High to very high information interest level 2 (*n* = 217)	COR (95% CI)	AOR (95% CI)	*P* value
Gender					0.492
Male	20 (6%)	34 (29.3%)	0.531 (0.979–2.953)	0.33 (0.542–3.568)	
Female	98 (10.1%)	183 (64.8%)	Referent	Referent	
Age					0.001*
< 40	3 (0.9%)	19 (5.7%)	1.846 (1.874–21.402)	2.105 (1.933–34.838)	0.004
40–49	16 (4.8%)	56 (16.7%)	1.253 (2.008–6.1)	1.719 (2.302–13.516)	0.000
50–59	37 (11%)	79 (23.6%)	0.759 (1.445–3.155)	1.314 (1.735–7.986)	0.001
60–69	23 (6.9%)	45 (13.4%)	0.671 (1.184–3.233)	1.291 (1.604–8.252)	0.002
≥ 70	39 (11.6%)	18 (5.4%)	Referent	Referent	-
Education					0.080
Illiterate	19 (5.7%)	7 (2.1%)	− 0.999 (0.155–0.876)	− 1.923 (0.27–0.79)	0.026
Elementary school	37 (11%)	41 (12.2%)	0.103 (0.711–1.728)	− 1.094 (0.075–1.504)	0.154
High school (Tawjihi)	29 (8.7%)	62 (18.5%)	0.76 (1.376–3.323)	− 0.867 (0.092–1.913)	0.262
Diploma	21(6.3%)	46 (13.7%)	0.784 (1.307–3.67)	− 0.942 (0.085–1.78)	0.224
University degree	11 (3.3%)	52 (15.5%)	1.553 (2.467–9.059)	− 0.230 (0.164–3.856)	0.775
Masters/PhD	1 (0.3%)	9 (2.7%)	Referent	Referent	-
Type of cancer					0.434
Breast	86 (25.7%)	169 (50.4%)	0.676 (1.516–2.548)	0.675 (0.329–4.188)	0.804
Colon	23 (6.9%)	36 (10.7%)	0.448 (0.928–2.641)	0.390 (0.220–2.016)	0.472
Rectum	9 (2.7%)	12 (3.6%)	Referent	Referent	-
Residence					0.556
North region	92 (27.5%)	172 (51.3%)	0.676 (1.516–2.548)	− 0.492 (0.164–2.273)	0.462
Middle region	24 (7.2%)	35 (10.4%)	0.448 (0.928–2.641)	− 0.733 (0.117–1.97)	0.308
South region	2 (6%)	10 (3%)	Referent	Referent	-
Monthly income (JD)	Very low to moderate level of interest in cancer information 1 (70)	High to very high information interest level 2 (137)			0.287
< 100 JD (< 140$)	8 (3.9%)	8 (3.9%)	0.000 (0.375–2.664)	− 0.776(0.108–1.969)	0.296
100–199 JD (140–279$)	13 (6.3%)	11(5.3%)	− 0.167(0.379–1.889)	− 1.324(0.076–0.933)	0.039
200–299 JD (280–419$)	18 (8.7%)	24(11.6%)	0.288 (0.724–2.457)	− 0.868(0.148–0.187)	0.102
300–499 JD (500–749$)	20 (9.7%)	43 (20.8%)	0.765 (1.2645–3.654)	− 0.448 (0.245–1.665)	0.359
≥ 500 JD (750$)	11 (5.3%)	51 (24.6%)	Referent	Referent	-

## Discussion

There has been minimal research on the information needs of Middle Eastern cancer survivors. The study investigated the information-seeking behaviours of breast and colorectal cancer patients in Jordan. Despite the fact that patients were in the survivorship phase of care, questionnaire scores on each dimension were consistently high, suggesting that patients were collectively under-informed. According to Budgell et al. [31], high information needs were found amongst cancer survivors. The persistent high level of interest in cancer-related information typologies may indicate a lack of high-quality educational materials and suboptimal information delivery methods that meet patients’ informational needs and expectations across the cancer trajectory. A systematic review of prior studies on the information needs on migrant Arab cancer patients identified the presence of unmet cancer information needs, in addition to physical needs and information on how to reduce caregiver dependency [20].

Patients prioritised information on disease stage, recurrence, and treatment options according to the findings. These findings corroborate prior research on cancer patients in Jordan [18, 32]. According to one study, Jordanian cancer patients were more concerned with information about the disease and medical procedures [32], whilst another found that patients had a strong desire for information about disease diagnosis, staging, and recurrence, as well as the possibility of cure and treatment adverse effects. Jordanian survivors appear to be the least interested in information on insurance and treatment costs. This was the item with the lowest score (mean was 3.37 out of 5). Financial and insurance domains were discovered to have no relationship with monthly income or employment status. This may be because the Jordanian government covers the costs of cancer treatment and there are no private or not-for-profit insurance schemes [33].

The primary source of cancer information was the treating physician, followed by the internet and family/friends/other survivors. Previously, similar outcomes were reported in conjunction with various sources, including radio/TV, pamphlets, and booklets [20, 31, 32]. Whilst nurses and pharmacists are crucial parts of patient education in western healthcare systems [19], Jordanian cancer survivors perceived them as a marginal source of information (6.9%), probably implying that multidisciplinary care for patients with breast and colorectal cancer is suboptimal. According to a recently published systematic review, studies examining patients’ information requirements have tended to focus on the diagnosis and treatment phases, with other stages of the cancer trajectory frequently disregarded [12].

The pattern of information-seeking behaviour in patients with various malignancies has been demonstrated to fluctuate over time [12, 34]. These distinctions are quantifiable (e.g. type of information sought, time spent in searching, search strategy, and comprehensiveness) [13].

Most survivors developed information needs early in the cancer continuum (e.g. within 2 months of diagnosis), and their information needs remained high beyond therapy [34]. In this study, regardless of the type of cancer, survivors developed their information needs at an early stage of the cancer continuum; few mentioned that they developed certain needs after recurrence. The reasons behind this informational inertia during recurrence could not be elucidated because our research did not consider the definitive diagnosis, stage, or severity of cancer; instead, treated cancer survivors were grouped together based on the type of cancer they had. These findings are discordant with previous studies in western societies suggesting that cancer patients with advanced disease or recurrence have greater needs. According to Squiers et al. [35], information needs for recurrence patients are more likely to include detailed treatment options and referrals to medical services. Additional cross-cultural research is required to understand how breast and colorectal cancer survivors’ needs vary according to their stage in the cancer trajectory.

A total proportion of ~ 16% of patients did not develop any information needs at any particular time; these findings are consistent with several studies in developed countries estimating that 10 to 30 of patients avoid information seeking as this might trigger emotions of anxiety, fear, or emotional distress or dissonance. Complex interactions may influence cancer patients’ information needs, which may be linked to demographics rather than time since diagnosis. A significant corpus of literature investigated the attributes and predictors that distinguish information seekers from avoiders [36–40]. The information needs of cancer patients vary based on their personality traits. Socio-demographic factors, such as education and income, as well as individual psychosocial factors, all have a significant impact on active information-seeking patterns [36–40].

In the binary logistic regression analysis, only age was found to be a statistically significant determinant for a high to very high information-seeking behaviour amongst Jordanian breast and colorectal cancer survivors. Younger patients aged 40–49 years were more likely to have a relatively high to very high degree of interest in overall cancer-related information typologies. These findings are partially consistent with the findings of Fiszer et al. [41], who conducted a systematic review of unmet supportive care needs of breast cancer patients and discovered that these needs clustered around psychological and information needs, but are influenced by individual characteristics such as demographic, psychosocial, and clinical factors. The authors concluded that younger age is systematically associated with greater information needs. Breast cancer is most common in women aged 55–64 (median age 62), with 8.3% diagnosed between 35 and 44, 19.7% between 45 and 54, and roughly 50% diagnosed between 55 and 74. Breast cancer was detected in Jordanian women at a median age of 50 years, with 30.6% diagnosed in the 40–49 age range [42]. As a result, it is impacting a greater number of younger women. Breast cancer accounted for 71.8% of our study population. This may explain why younger age was found to act as a predictor for informational needs. According to previous research, females and younger cancer survivors had higher supportive cancer needs [43, 44]. Colorectal cancer, on the other hand, occurs less commonly in young individuals and more frequently in the elderly [45]. All socio-demographic characteristics (age, income, education, and employment) were found to influence information requirements. However, only age was an independent predictor of high level of information needs.

## Conclusion and practical implications

Jordanian cancer survivors undergoing follow-up care and cancer surveillance were keenly interested to receive information about a variety of cancer topics during their cancer journey. Therefore, healthcare providers should be cognizant of the shifting trends of breast cancer incidence and fluctuation of information needs throughout cancer trajectory as most patients would continue requiring information at all stages. Cancer survivorship plans can be one of the most effective ways to provide information to cancer patients undergoing follow-up treatment. The National Cancer Institute (NCI) recommends supportive care and cancer survivorship planning for all cancer survivors. Consequently, this current study determined Jordan cancer survivors’ informational typologies and timings to support the widespread implementation of this recommendation. Research is needed to develop cost-effective delivery methods and ensure resource availability for information preparation and delivery [43, 44]. By recognising and prioritising the most critical information, patient contacts with the healthcare team can be made more meaningful and efficient. This study highlighted the typology of information prioritised by Jordanian survivors. This can help to establish a framework for patient education across the cancer care continuum [13].

Nevertheless, more longitudinal and prospective cross-cultural research is required to understand information needs of Arab cancer survivors and how they change throughout the stages of cancer continuum for facilitated real-time, personalised, and culturally relevant fulfilment of patients’ information requirements.

### Limitations

This study has limitations. To begin, the study population is limited to Jordanians with breast or colorectal cancer, with non-Jordanians or refugees being excluded. Second, we did not recognise specific cancer diagnoses, stages, or severity; rather, we classified treated cancer survivors broadly based on cancer type. To assess changes in cancer-related information typologies, a prospective longitudinal study that follows the same patients throughout their journey is preferred. A longitudinal design may also reduce bias due to illness complexity, severity, and course (advanced vs. early disease).

## Supplementary Information

Below is the link to the electronic supplementary material.

Supplementary file1 (DOCX 16 KB)

## Data Availability

The data used to support the findings of this study are available from the corresponding author upon reasonable request.

## Code availability

Software application and coding data are available from the corresponding author upon reasonable request.

## References

[1] Sung H, Ferlay J, Siegel RL, Laversanne M, Soerjomataram I, Jemal A, Bray F (2021). Global Cancer Statistics 2020: GLOBOCAN estimates of incidence and mortality worldwide for 36 cancers in 185 countries. CA Cancer J Clin.

[2] World Health Organisation (2020) International Agency for Research on Cancer; Jordan Globocan. https://gco.iarc.fr/today/data/factsheets/populations/400-jordan-fact-sheets.pdf; Accessed on 28 Jan 2022

[3] National Coalition for Cancer Survivorship. http://www.canceradvocacy.org/; Accessed on 28 Jan 2022

[4] Marzorati C, Riva S, Pravettoni G (2017). Who is a cancer survivor? A systematic review of published definitions. J Cancer Educ.

[5] Moore HCF (2020). Breast cancer survivorship. Semin Oncol.

[6] Mosher CE, Winger JG, Given BA, Helft PR, O’Neil BH (2016). Mental health outcomes during colorectal cancer survivorship: a review of the literature. Psychooncology.

[7] Groen WG, Kuijpers W, Oldenburg HS, Wouters MW, Aaronson NK, van Harten WH (2015). Empowerment of cancer survivors through information technology: an integrative review. J Med Internet Res.

[8] Ryhänen AM, Siekkinen M, Rankinen S, Korvenranta H, Leino-Kilpi H (2010). The effects of Internet or interactive computer-based patient education in the field of breast cancer: a systematic literature review. Patient Educ Couns.

[9] Warren E, Footman K, Tinelli M, McKee M, Knai C (2014). Do cancer-specific websites meet patient’s information needs?. Patient Educ Couns.

[10] Bergenmar M, Johansson H, Sharp L (2014). Patients’ perception of information after completion of adjuvant radiotherapy for breast cancer. Eur J Oncol Nurs.

[11] Harrison JD, Young JM, Price MA, Butow PN, Solomon MJ (2009). What are the unmet supportive care needs of people with cancer? A systematic review. Support Care Cancer.

[12] Rutten LJ, Arora NK, Bakos AD, Aziz N, Rowland J. Information needs and sources of information among cancer patients: a systematic review of research (1980–2003). Patient 10.1016/j.pec.2004.06.006 15893206

[13] Tariman JD, Doorenbos A, Schepp KG, Singhal S, Berry DL (2014) Information needs priorities in patients diagnosed with cancer: a systematic review. J Adv Pract Oncol. 2014(5):115–122 PMC4042668 24910808

[14] Almyroudi A, Degner LF, Paika V, Pavlidis N, Hyphantis T (2011). Decision-making preferences and information needs among Greek breast cancer patients. Psychooncology.

[15] Kimiafar K, Sarbaz M, Shahid Sales S, Esmaeili M, Javame GZ (2016). Breast cancer patients’ information needs and information-seeking behavior in a developing country. Breast.

[16] Sheikhtaheri A, Nahvijou A, Mashoof E (2020). Information needs of women with breast cancer: a review of the literature. Frontiers in Health Informatics.

[17] Li PW, So WK, Fong DY, Lui LY, Lo JC, Lau SF (2011). The information needs of breast cancer patients in Hong Kong and their levels of satisfaction with the provision of information. Cancer Nurs..

[18] Obeidat R, Khrais HI (2015). Information needs and disclosure preferences among Jordanian women diagnosed with breast cancer. J Cancer Educ.

[19] Abi Nader E, Kourie HR, Ghosn M, Karak FE, Kattan J, Chahine G, Nasr F (2016). Informational needs of women with breast cancer treated with chemotherapy. Asian Pac J Cancer Prev.

[20] Alananzeh I, Levesque J, Kwok C, Everett B (2016). Integrative review of the supportive care needs of Arab people affected by cancer. Asia Pac J Oncol Nurs..

[21] Findik UY (2017). The information needs of women who have undergone breast cancer surgery in the west of Turkey. J Cancer Educ.

[22] Krejcie RV, Morgan DW (1970). Determining sample size for research activities. Educ Psychol Measur.

[23] Beaver K, Bogg J, Luker KA (1999). Decision-making role preferences and information needs: a comparison of colorectal and breast cancer. Health Expect.

[24] Tian L, Cao X, Feng X (2019). Evaluation of psychometric properties of needs assessment tools in cancer patients: a systematic literature review. PLoS ONE.

[25] Kassianos AP, Raats MM, Gage H (2016). An exploratory study on the information needs of prostate cancer patients and their partners. Health Psychol Res.

[26] Faller H, Koch U, Brähler E, Härter M, Keller M, Schulz H, Wegscheider K, Weis J, Boehncke A, Hund B, Reuter K, Richard M, Sehner S, Szalai C, Wittchen HU, Mehnert A (2016). Satisfaction with information and unmet information needs in men and women with cancer. J Cancer Surviv.

[27] Harpe SE (2015). How to analyze Likert and other rating scale data. Curr Pharm Teach Learn.

[28] Hassan ZA, Schattner P, Mazza D (2006). Doing a pilot study: why is it essential?. Malays Fam Physician.

[29] Williams A (2003). How to write and analyse a questionnaire. J Orthod..

[30] WHODAS 2.0 Translation package https://terrance.who.int/mediacentre/data/WHODAS/Guidelines/WHODAS%202.0%20Translation%20guidelines.pdf. Accessed 31 Jan 2022

[31] Shea-Budgell MA, Kostaras X, Myhill KP, Hagen NA (2014). Information needs and sources of information for patients during cancer follow-up. Curr Oncol.

[32] Al QM (2014). Jordanian cancer patients’ information needs and information-seeking behaviour: a descriptive study. Eur J Oncol Nurs.

[33] Abdel-Razeq H, Attiga F, Mansour A (2015). Cancer care in Jordan. Hematol Oncol Stem Cell Ther.

[34] Mistry A, Wilson S, Priestman T, Damery S, Haque M (2010). How do the information needs of cancer patients differ at different stages of the cancer journey? A cross-sectional survey. JRSM Short Rep.

[35] Squiers L, Finney Rutten LJ, Treiman K, Bright MA, Hesse B (2005). Cancer patients’ information needs across the cancer care continuum: evidence from the cancer information service. J Health Commun.

[36] Lambert SD, Loiselle CG (2007). Health information seeking behavior. Qual Health Res.

[37] Case DO, Andrews JE, Johnson JD, Allard SL (2005). Avoiding versus seeking: the relationship of information seeking to avoidance, blunting, coping, dissonance, and related concepts. J Med Libr Assoc.

[38] Ramanadhan S, Viswanath K (2006). Health and the information nonseeker: a profile. Health Commun.

[39] Czaja R, Manfredi C, Price J (2003). The determinants and consequences of information seeking among cancer patients. J Health Commun..

[40] Miller SM (1987). Monitoring and blunting: validation of a questionnaire to assess styles of information seeking under threat. J Pers Soc Psychol.

[41] Fiszer C, Dolbeault S, Sultan S, Brédart A (2014). Prevalence, intensity, and predictors of the supportive care needs of women diagnosed with breast cancer: a systematic review. Psychooncology.

[42] Ismail SI, Soubani M, Nimri JM, Al-Zeer AH (2013). Cancer incidence in Jordan from 1996 to 2009–a comprehensive study. Asian Pac J Cancer Prev.

[43] Haq R, Heus L, Baker NA, Dastur D, Leung FH, Leung E, Li B, Vu K, Parsons JA (2013). Designing a multifaceted survivorship care plan to meet the information and communication needs of breast cancer patients and their family physicians: results of a qualitative pilot study. BMC Med Inform Decis Mak.

[44] Singh-Carlson S, Wong E, Martin L, Nguyen SK (2013). Breast cancer survivorship and South Asian women: understanding about the follow-up care plan and perspectives and preferences for information post treatment. Curr Oncol.

[45] National Cancer Institute, fact sheets: https://seer.cancer.gov/statfacts/html/breast.html. Accessed on 30 Jan 2022

