# Supplementary material for: Informational needs and predictors of Jordanian breast and colorectal cancer survivors: a national cross-sectional study
Source: Support Care Cancer. 2022 May 10;30(8):6827–37. doi: 10.1007/s00520-022-07110-6 (PMC9213385; doi:10.1007/s00520-022-07110-6)
Supplement: Supplementary file 1 — Supplementary file1 (DOCX 16 KB) [file 520_2022_7110_MOESM1_ESM.docx]

Supplementary Tables & Figures

Supplementary Table 1: Response proportions of the sample for each type of cancer, Jordan 2020

|  | Sample response proportions | | |
| --- | --- | --- | --- |
| Type of cancer | Eligible Sample Size of Population Frame | Responded Study Sample | |
|  | N | n | % |
| Breast | 303 | 255 | 84.16 |
| Colon | 80 | 59 | 73.8 |
| Rectum | 26 | 21 | 80.8 |
| Total | **409 | 335 | 81.9 |

Supplementary Table 2: Results for overall sample proportions according to causes of non-response of eligible sample.

| Causes of Non-Response of Eligible Sample | n | % |
| --- | --- | --- |
| Refused to answer | 21 | 5. 1 |
| Died during collection of data | 5 | 0.01 |
| Telephone not in use | 35 | 8.55 |
| Telephone disconnected by the company | 7 | 1.7 |
| Telephone number not at all for the patient in the sample | 4 | 0.97 |
| The patient lived outside Jordan | 2 | 0.48 |
| Responded | 335 | 81.9 |

Supplementary Table 3: Sources sought by cancer survivors for cancer related information, Jordan 2020

| **Sources used for cancer-related information n=335** | **n (%) *** |
| --- | --- |
| Treating Doctor | 285 (85.1) |
| HCPs (e.g., pharmacist, nurses) | 23 (6.9) |
| Internet | 156 (46.6) |
| Family/friends/other patients’ advice | 40 (11.9) |
| Magazines/journals/books | 37 (11.9) |
| Information leaflets | 30 (9.0) |
| Radio/TV | 30 (9.0) |
| Courses/seminars | 27 (8.1) |

*Percentages and totals based on respondents; this adds to more than 100% as multiple responses were allowed.
